# Perceived barriers to the implementation of Isoniazid preventive therapy for people living with HIV in resource constrained settings: a qualitative study

**DOI:** 10.11604/pamj.2014.17.26.2641

**Published:** 2014-01-17

**Authors:** Mesele Mindachew, Amare Deribew, Peter Memiah, Sibhatu Biadgilign

**Affiliations:** 1 Department of General Public Health, College of Public Health and Medical Science, Jimma University, Ethiopia; 2 Department of Epidemiology, College of Public Health and Medical Science, Jimma University, Jimma, Ethiopia; 3 University of Maryland School of Medicine-Institute of Human Virology, Baltimore, MD

**Keywords:** Isoniazid preventive therapy, People Living with HIV, TB

## Abstract

**Introduction:**

Isoniazid preventive therapy (IPT) reduces the risk of active TB. IPT is a key public health intervention for the prevention of TB among people living with HIV and has been recommended as part of a comprehensive HIV and AIDS care strategy. However, its implementation has been very slow and has been impeded by several barriers. Objective: The Objective of the study is to assess the perceived barriers to the implementation of Isoniazid preventive therapy for people living with HIV in resource constrained settings in Addis Ababa, Ethiopia in 2010.

**Methods:**

A qualitative study using a semi-structured interviewed guide was used for the in-depth interview. A total of 12 key informants including ART Nurse, counselors and coordinators found in four hospitals were included in the interview. Each session of the in-depth interview was recorded via audio tape and detailed notes. The interview was transcribed verbatim. The data was analyzed manually.

**Results:**

The findings revealed that poor patient adherence was a major factor; with the following issues cited as the reasons for poor adherence; forgetfulness; lack of understanding of condition and patient non- disclosure of HIV sero-status leading to insubstantial social support; underlying mental health issues resulting in missed or irregular patient appointments; weak patient/healthcare provider relationship due to limited quality interaction; lack of patient information, patient empowerment and proper counseling on IPT; and the deficient reinforcement by health officials and other stakeholders on the significance of IPT medication adherence as a critical for positive health outcomes.

**Conclusion:**

Uptake of the implementation of IPT is facing a challenge in resource limited settings. This recalled provision of training/capacity building and awareness creation mechanism for the health workers, facilitating disclosure and social support for the patients is recommended.

## Introduction

The estimates of the global burden of disease caused by TB in 2009 are 9.4 million incident cases, 0.38 million deaths among HIV-positive people. An estimated 11-13% of incident cases were HIV-positive [1]. Most of the estimated number of cases in 2009 occurred in Africa (30%). The 22 High burden countries (HBCs) that have received particular attention at the global level since 2000 account for 81% of all estimated cases worldwide. Ethiopian ranks seventh in the list. In 2009, 26% of TB patients knew their HIV status (up from 22% in 2008), including 53% of patients in the African Region [1]. Tuberculosis (TB) has reemerged as a major threat to global public health. Its incidence is rising, particularly in countries with a high HIV prevalence [2]. HIV-infected persons have an increased risk for reactivated latent TB infection [3].

Tuberculosis (TB) is the most frequent life-threatening opportunistic disease among people living with HIV and remains a leading cause of mortality, even among persons receiving ART [4]. In 1998, the WHO and the United Nations Joint Program on HIV/AIDS (UNAIDS) issued a new IPT policy with 6 key steps as a part of the package of care for people living with HIV [5]. To prevent TB, almost 80 000 people living with HIV were provided with isoniazid preventive therapy. This is an increase from previous years, but still represents less than 1% of the estimated number of people living with HIV worldwide [1]. Isoniazid preventive therapy (IPT) reduces the risk of active TB by approximately 33% overall [6]. IPT is a key public health intervention for the prevention of TB among people living with HIV and has been recommended since 1998 by WHO and the Joint United Nations Programme on HIV/AIDS (UNAIDS) as part of a comprehensive HIV and AIDS care strategy [7]. However, its implementation has been very slow and has been impeded by several barriers including lack of an accepted approach to exclude active TB disease and restricted access to isoniazid for fear of developing drug resistance. By the end of 2009, globally only 85 000 people living with HIV received IPT[1].

Despite WHO guidelines recommending IPT as part of routine HIV care, there has been little IPT implementation at country level [8]. However, in resource-limited settings these benefits are rarely realized. Obstacles include cost, interrupted drug supplies and suboptimal adherence [9, 10]. The greatest obstacle to successful LTBI treatment programs is poor patient adherence [11]. To enable all those infected by HIV to benefit from these life-saving interventions, it is imperative that barriers to adherence to IPT be urgently and innovatively addressed. The objective of this study was to asses an in-depth investigation of the perceived barriers to the implementation of isoniazid preventive therapy for people living with HIV in resource constrained settings.

## Methods

### Study setting

The study was conducted in Yekatit 12, Zewditu, Gandi and Minilik Hospitals, of Addis Ababa city from February 1- March 30, 2010. At present approximately 1800 adults are receiving IPT in Ethiopia. Among the study hospitals, Minilik is a central generalized referral hospital and are under Federal Ministry of Health. Yekatit 12 and Zewditu Hospitals are under Addis Ababa Regional Health Bureau, known to provide service to serve most of the patients on IPT follow up. Administratively, each of the hospitals is divided into sub city to serve clients from each sub city. Zewditu hospital gives service for Kirkose and Akaki sub cities. Yekatite 12 gives service for Yeka and Addis ketema sub cities. In the study period, according to the report obtained from the registration record of the ART unit in the respective hospitals, 1303 adults were found in the study site that were on IPT and on follow-up.

### Design and participants

For this qualitative study, those interviewed were health care workers who were working on counseling of patients on the importance of drug adherence and how to recognize common adverse drug reactions associated with INH and individuals involved in, or supporting the provision of treatment and care for HIV infected clients. Purposive sampling technique was carried out to select the participants based on exposure to their patients in all circumstances, their proximity to appreciate the problems of adherence and capability to suggest interventions mechanisms for improving adherence to IPT and adherence status of their patient particularly adherence counselor, ART nurses and ART coordinators was enrolled for in-depth interview and their number were determined by saturation of ideas generated by the participants in successive interviews until their ideas become repetitive and redundant so that no new information can be gathered by further data collection. In addition, these participants were expected to present for follow up during the data collection period. In depth interview was undertaken with the health care workers with diverse social economic and demographic characteristics. Participants were eligible for the study if they expressed willingness to share their views on patient's adherence to Isoniazid (INH).

### Data collection

In-depth interviews were conducted with health workers including counselors and coordinators by the principal investigator (PI). An interviewer guide was used for the in-depth interview. In undertaking the interview sessions, a guide containing the following areas was used: Knowledge on the disease and its Treatment, Factors that influence patient adherence to IPT, Personal perspectives to the medication and Barriers and/or facilitators of patient adherence to treatment. Interviews were conducted in a hospital set up that was convenient for key informants. Field notes were written at the conclusion of each interview and all tapes were transcribed. The data collection method was checklist guided in-depth interviewing technique with health care provider who has close contact with the patient under follow up in ART clinic. After the subjects were asked for their voluntary participation, the conversation was tape recorded and further scrutinized to discover recurrent patterns and saturation of ideas before the themes was identified and interpreted. Ethical clearance was obtained from Jimma University and Addis Ababa Health Bureau.

### Data analysis

The data processing and analysis procedures were begun on the first day after interview. On a daily basis, data were processed, and patterns were identified based on saturation of ideas throughout the study. The data were fully transcribed and analyzed manually. We analyzed the interview data using a thematic content analysis approach. This approach is a comparative process by which the various accounts gathered are compared with each other to classify those “themes” that recur or are common in the data set [12]. All transcripts were read several times by the investigators separately to bring out the main ideas. Later the data was reviewed and combined into broader categories in terms of key variables; it was then ordered, reduced and classified or coded (sorted out), displayed, summarized and finally interpreted.

## Results

The purpose of qualitative research was to obtain in-depth responses concerning what people think and feel concerning a particular area of interest. To this end, the research team employed an in-depth interview with health care providers; namely ART nurse, adherence counselors and ART coordinators. A total of 12 key informants were purposively selected and volunteered to participate. A total of 12 patients, 7(58%) females were interviewed. Ages ranged from 27 to 48 years, with a mean age of 34 years. Proportional allocation was made according to their profession (Table 1).

**Table 1:** Socio-demographic attributes of the respondents participated in Addis Ababa hospitals, 2010

Respondent	Age	Sex	Profession/Responsibilities
R1	34	F	ART nurse
R2	28	M	ART coordinator
R3	40	F	Adherence counselor
R4	29	M	Adherence counselor
R5	33	F	ART nurse
R6	48	F	ART coordinator
R7	30	F	ART nurse
R8	29	M	ART nurse
R9	38	M	Adherence counselor
R10	45	M	ART coordinator
R11	31	F	Adherence counselor
R12	27	F	ART coordinator

Three major themes influencing IPT uptake and adherence identified includes: - A) factors related with patient provider interaction, B) patient related factors, C) socio-environmental factors, barriers and IPT drugs.

### Factors related with patient provider interaction

The first question asked under this theme was to describe patient-provider relation in relation to barriers for patient's adherence to IPT. Except four participants the rest indicated that they had good relation with the patient, most of the participant said that the patients had good experience to attend regular and follow up appointments, they accept and practice as advised, they ask questions when faced with confusion related with the regimen, they ask for help when they develop side effects, but in contrast to this a 31 years old female adherence counselor said

*“I was very much disappointed with some patients due the fact that they completely forget what I have told to them when they came in the subsequent visit missing some of their pills, even they will not be interested to talk about issues related with IPT and the most frequently reported reason by the client was they don't want to stay for long time talking about regimens/treatment other than ART, they give much emphasis to ART than IPT and cotrimoxazole prophylaxis”*.

In this regard 48 years old ART coordinator also emphasized on the provision of necessary information to the client, he said,

*“We are not motivated enough to provide information to the patient, I myself were not doing that because the higher officials are not giving much emphasis to the regimen, even I haven't been trained, here great emphasis was still given to the ART than other TB/HIV collaborative activities, and that is why we were not providing enough information to the client?”*.

In relation to this one female ART nurse also emphasized

*“ We can't discuss IPT due to lack of enough time and heavy patient load, patients are frequently complaining about long waiting time to get the service”*.

Similarly a 29 male ART nurse from other hospital said also,

*“In my opinion, before the initiation of IPT, patients must know for how long, why, when and how they should take the prescribed pills, then the patient will be committed to taking the drug, but what was really happening in our case is that we only told them just the highlight which is not sufficient enough to make them develop belief in the regimen and to be adherent. ”*.

### Patient related factors

Under this theme majority of the respondents relate this group of factors as a barrier and facilitator of adherence. When they were asked to evaluate the knowledge of patients about the disease and treatment, most of them raised similar issues. As they reported, though the client didn't have deep knowledge concerning the regimen and the importance of adherence, in all hospitals the client had good level of adherence In line with this a 45 years old Medical doctor who functions as the ART clinic coordinator said,

*“As estimated by ART nurses most of our clients have good levels of adherence but which doesn't really indicate that they were well informed about the drug because as I myself communicate to some of the client they have no idea why they are taking the drug and they say they are simply taking it as part of the ART drugs with no relation to tuberculosis treatment even they didn't know why they are taking the drug they simply take it as part of ART drug, they didn't relate it to tuberculosis”*.

In contrast to the above idea a 38 years old male adherence counselor from other hospital said:

*“Our clients have good level of adherence, because we are providing sufficient information to them concerning the details of the regimen, the importance of adherence and other related issues. In addition to our staff who are working in ART clinic, we have got NGO partners working on TB-HIV collaborative activities and at the same time they are providing us with technical and material support so that to enhance the efficacy of the treatment for both ART and IPT”*.

The other most frequently mentioned barriers of adherence to IPT as most of the respondent indicated the patient have mental health issues which make reluctant to attend follow-up and clinical appointment regularly, to stick to the scheduled time of drug administration(mental health issues directly or indirectly related to the disease) and which might also affect their understanding of the regimen, the disease and their understanding of all the instructions and information given by health care provider.

In line with this, a 40 years old adherence counselor from one of the hospitals said:

*“A patient with disturbed emotion and who have developed side effect at some time during the course of treatment are more likely to miss or to be non adherent than those who are not ”*.

As raised by most of the key informant, the other important factor which had great emphasis was that the patients are not willing to disclose their sero-status to their family members or to others who are close to them and as indicated by the key informants. Most of the patients did that because they claimed fear of disturbing or losing the normal relationships they had with their families and within their communities as major reasons for not disclosing their sero-status (fear of stigma and discrimination) and due to this they lack the courage to take the drugs in public forcing them to miss some of their prescribed medication.

### Socio-environmental factors

When they were asked about the requirements/recommendation to enhance adherence status of the patient, the efficacy of the therapy, all participants suggested that social support from a family, community, NGOs and others were important factor in assisting them to adhere to their drug regimen; and also it is expected that the government should provide more emphasis to this new program, all heath care providers should be well trained and take responsibilities to give care, support and information concerning the drug and the disease to the patient so that to promote and maintain maximum adherence.

## Discussion

In developing countries, preventive therapy has not received much emphasis [13]. However, with the advent of HIV infection, TB has emerged as a major opportunistic infection, particularly in developing countries. HIV infection is the single greatest risk factor for the reactivation of latent mycobacterial infection [14]. In the 2009 WHO TB Control Report, the WHO estimates that fewer than 0.5% of HIV-infected persons Worldwide have received IPT [15]. In our study, most of the respondents, the heath care workers, demonstrated that they didn't have the capacity to provide adequate information and prescription to the patients as they didn't get the training. The finding is similar to other studies. The lack of familiarity and confidence in IPT prescribing was evidenced not only by variable and inconsistent prescribing practices, but by healthcare workers? own admission that they were unclear about its benefits. In a programme that has promoted IPT since its inception in 1999, this is even more remarkable, and policy clarification and dissemination of accurate information is required[16]. Prior to implementation of THRio, physician knowledge about the national recommendations for TB prevention in HIV patients was limited [17]. In other studies clinicians are also concerned about toxicity of isoniazid and maintaining adherence to the IPT regimen [18]. In Tanzania, the reasons provided by the completers for IPT/ decision to complete IPT were fear of TB, understanding of the importance of IPT, and fear of TB and HIV complications [19]. Likewise, Compared to non-completers, completers were more likely to be highly motivated to adhere [11]. So the Application of intensive patient education, enablers may improve adherence[20, 21]. The Constraints mentioned for barrier for implementation were, among others: limited motivation and knowledge by counselors to discuss TB issues during HIV pre- and post test Counseling [22]. Among physicians who did not provide IPT, the most common reason were patient's poor adherence (24.8%), IPT might induce the occurrence of Isoniazid-resistant TB cases (20.2%), concern about side effect of Isoniazid (12.8%), and they felt that IPT was not beneficial (6.2%)[23]. The high rate of completion was due to a number of factors, including personal experience with TB, knowledge gained from counseling, family approval and proximity to the clinic[19]. One of the factors that the health care provider revealed about their patients is that they forget to take their pill and keep appointments. Similarly in there, are reported reasons for poor adherence including lack of transport, being away from home, loss of drugs and forgetfulness[24] and patients who report they sometimes forget to take their medication were more likely to have a negative urine test [25]. This was also previously reported as a predictor in another study of patients on INH for LTBI[26]. In a similar fashion there was inadequate patient-provider relationship as was evidenced from our study due to high patient volumes. A study in Brazil showed that although physicians at the public health units say they are too busy, tense and solitary, they evaluated their health units very positively[17].

One of the factors that identified for barriers for the implementation was HIV sero status's disclosure. Other study finding supports this finding. Five of the six patients in the group who completed TBPT had disclosed to someone who was able to support them emotionally and financially. However, the fear of rejection and stigmatization prevented people from disclosing their HIV status [27]. A preventive therapy service requires supervision of clients for adherence to therapy. Convincing patients who are essentially well to take medication for an extended period is difficult. This requires patient education which is time consuming and may not lead to sustained adherence[13]. In rural South Africa study, Barriers to adherence included fear of stigmatization, the belief that HIV is incurable, and a reluctance to take medication in the absence of symptoms but disclosure of HIV status, social and family support, and a supportive clinic environment positively influenced adherence [27]. In the same study important barriers to adherence noted among all groups interviewed include fear of stigmatization in relation to their HIV status, lack of money for food and transport, the belief that HIV is incurable by Western medicine[27].

Along with this, fear of stigma and discrimination incur a lot for disclosure of the status and for this reason they lack courage to take their pills in public. Similar studies demonstrated and support our finding. Those believing they have a higher above average chance of getting active TB without INH were more likely non-adherent. Patients may perceive stigma or conceal their condition and its treatment, which could interfere with adherence. Alternatively, knowledge of the danger of TB may motivate them to share or sell their INH to others who are also at risk[25]. In South Africa five of the six patients in the group who completed TBPT had disclosed to someone who was able to support them emotionally and financially. However, the fear of rejection and stigmatization prevented people from disclosing their HIV status and Lack of social and family support was identified as adversely affecting adherence. Similarly the presence of supportive nurses in the clinic also appears to play a role in the decision to regularly visit the clinic and pick up medication[27]. At an ART pilot initiative in South Africa, good adherence was attributed to even more rigorous enrolment criteria, including, the requirement to attend support groups, openness about HIV status, and proven adherence to other chronic disease medications[28]. This is similar to qualitative research done in Thailand where those who completed TBPT cited the acceptance of their HIV status, their concern for their families? well-being, and good relationships with health care providers as promoting good adherence[29].

## Conclusion

The study demonstrated that uptake of the implementation of IPT is facing a challenge in resource limited settings. As much as the patients are to play a part in ensuring adherence to their treatment plan, providers must become a part of the solution. By identifying barriers to adherence, steps to improve adherence rates can be put into place. These include collaboration, open communication between healthcare professionals and patients regarding medication regimens, as well as education provided to patients about the benefits of disclosure, medication adherence and adherence to clinic appointments. This study also highlights the recalled provision of capacity building and awareness creation mechanism by health officials ensuring proper integration of the health system to support good adherence patterns for patients on IPT.
